# The Role of an Integrated Multidisciplinary Clinic in the Management of Patients with Cutaneous Lymphoma

**DOI:** 10.3389/fonc.2015.00136

**Published:** 2015-06-17

**Authors:** Kelly H. Tyler, Bradley M. Haverkos, Justin Hastings, Eileen Hu, Ramez Philips, Alejandro A. Gru, Meng Xu Welliver, Anjali Mishra, Henry K. Wong, Pierluigi Porcu

**Affiliations:** ^1^Division of Dermatology, The Ohio State University, Columbus, OH, USA; ^2^Division of Hematology, The Ohio State University, Columbus, OH, USA; ^3^Cutaneous Lymphoma Program, The Ohio State University, Columbus, OH, USA; ^4^School of Medicine, The Ohio State University, Columbus, OH, USA; ^5^Department of Pathology, The Ohio State University, Columbus, OH, USA; ^6^Department of Radiation Oncology, The Ohio State University, Columbus, OH, USA; ^7^Comprehensive Cancer Center, The Ohio State University, Columbus, OH, USA

**Keywords:** hematology/oncology, dermatology, medical education, research, multimodality

## Abstract

The clinical benefit of a multidisciplinary clinic practice model has been well described in a variety of medical specialties and cancer types. It proves particularly valuable when an integrated team is needed to optimally manage patients with rare or complex neoplasms. However, the ideal implementation of an integrated multidisciplinary care program for translational research and education has not been well reported. Herein, we propose how a multimodality cutaneous lymphoma (CL) clinic model can optimally manage CL patients. We offer our perspective on this model as an efficient means for delivering patient care, a continuing education resource for referring physicians, a conduit for translational and clinical research, and an educational tool for medical students, house staff, and fellows.

## Introduction

In today’s changing healthcare environment, providing efficient care in a cost-effective manner is an urgent priority. An integrated team approach resulting in improved health outcomes has been reported in various specialties (1–6). A multidisciplinary (or multimodality) clinic is defined as a group of health care professionals who have cognitive and procedural expertise in different areas of care delivery and can efficiently manage complex medical conditions. Studies of disease-focused multidisciplinary clinics have shown improved outcomes (7–9), whereby reduced mortality has been well documented in both meta-analyses and randomized trials (10–12). Additionally, economic analyses have demonstrated their cost-effective approach for complex patients and diseases (1, 2).

Cutaneous lymphomas (CLs) are a rare family of extranodal non-Hodgkin’s lymphomas, a majority of which (~70%) (13) are cutaneous T-cell lymphomas (CTCLs). CTCLs are difficult to diagnose in early stage but are often indolent neoplasms characterized by a good response to skin-directed therapies. However, many patients progress to more advanced stages (14) and need systemic therapy (15). One of the challenges in CTCL is the timing and coordination of the transition from skin-directed therapy to combined-modality therapy (skin directed + systemic). The transition itself is logistically complex, as it very often implies a shift from dermatology-driven to oncology-driven care. Communication among specialists about the appropriate goals of care and how to best execute the treatment plan is far from adequate. Patients are often confused about who is in charge of their care, resulting in poor satisfaction and outcomes.

The value of a multidisciplinary care model in CL has been consistently emphasized (16–18), but a blueprint for the design and implementation of such a practice model is not easy to find. The importance of a collaborative approach for advancing research has also been underscored (19). In 2009, we designed and established a multidisciplinary cutaneous lymphoma clinic (MCLC) at the Ohio State University Comprehensive Cancer Center (OSUCCC) with the goal of creating a novel integrated care delivery model for this patient population in central Ohio as well as a resource for referring physicians. Additional goals included the creation of a translational research program in CTCL and establishing the MCLC as a device to teach the principles and practice of translational cancer research and multidisciplinary care.

## The MCLC Model

**Figure 1:**
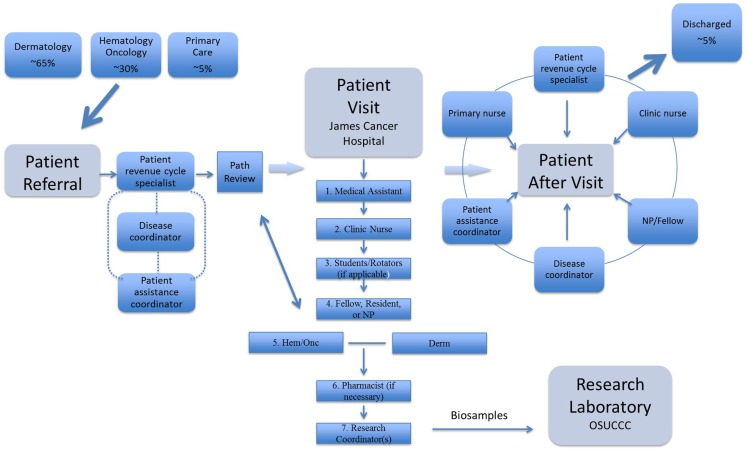
**Patients are referred from dermatology, medical oncology, and primary care**. After patient referral, the patient revenue specialist requests outside pathology to be reviewed at our institution, requests medical records, and schedules appointment time. Our expert cutaneous hematopathologist reviews outside pathology prior to initial patient visit and then will on some occasions see the patient during first visit. Patients in need of financial assistance are handled by our patient assistance coordinator. The disease coordinator acts to ensure the initial patient referral is following a smooth transition. Then, at time of the patient visit, the medical assistant greets and escorts the patient into the room. The new patient is checked-in by the clinic nurse who collects basic symptoms, past-medical history, social history, family history, medication list, and allergies. The patient is then seen by medical students/residents and/or our clinic fellow/NP. Following a detailed discussion by the NP/fellow, an expert cutaneous lymphoma dermatologist and hematologist/oncologist jointly complete the visit and finalize the plan. Our pharmacist is always available for further questions on new medications and always counsels patients who are about to undergo new chemotherapy. The initial patient visit is completed by our research coordinator(s) who consent patients for our lymphoma registry and tissue repository. After patient visit and discharge, the multimodality team continues to work together to provide follow up care.

Patients are referred to a centralized lymphoma scheduling office, whose staff is trained to triage patients to the MCLC, located at the James Cancer Hospital of the OSUCCC (Figure 1 and Table 1). At intake, research coordinators consent patients for an IRB-approved lymphoma database and biorepository; all archived samples are therefore clinically annotated. The dermatologist and hematologist–oncologist see the patient together, collaborating to formulate the assessment and plan and jointly addressing all questions and concerns. A clinical pharmacist advises the patients on drugs prescribed during the visit. A patient assistance coordinator ensures the patients’ access to prescribed treatments. The MCLC nurse provides patients with an after-visit summary, including contact information for all resources, team members, and educational tools. In all cases, biopsies are reviewed by an expert CL pathologist. Extra-corporeal photopheresis (ECP), radiation therapy (RT), and bone marrow transplantation (BMT) are closely coordinated with our colleagues. Patient satisfaction data are collected after each visit.

**Table 1:** **Essential elements of a multimodality cutaneous lymphoma clinic**.

Job title	Number	Function
Heme/Onc Physician	1	Formulate patient’s management plan in coordination with the dermatologist
Dermatology Physician	1	Formulate patient’s management plan in coordination with the hematologist/oncologist
Disease Coordinator	1	Patient care coordination, determine master schedule
Primary Nurse	1	Triage patient calls, prior authorizations, patient education, patient care coordination
Clinic Nurse	1 or multiple	Update patient history, draw blood, triage patient calls
Medical Assistant	1 or multiple	Room patients, assist with biopsies
Pharmacist	1	Educate patients on new medications, assist with prior authorizations, work with patient assistance coordinator on financial assistance for medications
Patient Assistance Coordinator	1	Determine patient eligibility for financial assistance and file assistance applications
Nurse Practitioner	1	See patients with physicians, laboratory follow-up, medication refills, manage patient calls
Research Coordinator	1	Determine patient eligibility for biorepository, lymphoma database, consent patients
Fellow/Resident	1 or multiple	See patients with physicians, laboratory follow-up, medication refills, manage patient calls, research
Pathologist	1	Review outside pathology, interpret in-house biopsies, interact with Heme/Onc and Derm physicians
Students/Rotators	2 per clinic	See patients with the fellow and shadow physicians, research
Patient Revenue Cycle Specialist	1 or multiple	Makes appointment for new patient referrals. Requested outside records and pathology for review. After appointment, schedules return visits

## Benefits of a Multidisciplinary Cutaneous Lymphoma Clinic

### Efficiency in clinical care delivery

The integrated multidisciplinary care model consolidates the expert advice of more than one specialist into a single outpatient encounter. Furthermore, an entire team of specialized nurses, pharmacists, and support staff is available to answer questions and provide resources, all in one location (Table 1). A team approach by a dermatologist and hematologist/oncologist can be achieved with separate office visits and frequent communication; however, management is more efficient and effective with an integrated approach. When two specialists with extensive experience in the recognition and management of the disease can directly communicate and make treatment decisions at a single point in time, patient satisfaction and outcomes are superior. Furthermore, this integrated practice model produces a high level of overlap in clinical expertise between dermatology and hematology–oncology, allowing each practitioner to efficiently and confidently step in when the other is away, and creating support staff familiar with all aspects of care.

A “one-stop” approach is particularly welcomed by patients traveling from out of state but is also valuable in longitudinal care. In total, 1256 patients (75% CTCL, 18% CBCL, 7% other), 365 of them new consultations, were seen at the MCLC over the past 5 years. About two-thirds were referred with an established diagnosis and one-third for a diagnostic work-up. For more than 50%, the MCLC is the primary provider and care-coordinator. Less than 5% of the patients were discharged from the clinic based on having a diagnosis other than CL. Patient satisfaction data, as measured by physician performance, indicate that the MCLC consistently ranks above its peers in the Internal Medicine Department (mean score 98.4 out of 100) for amount of time spent with the patient, physician’s skill and knowledge, and overall quality of care.

### A tool for referring physicians

Most dermatologists and hematologist/oncologists do not routinely see or manage CL, a disease where decision-making can often be challenging. We regularly see patients who were treated with chemotherapy when a skin-directed approach would have been adequate, or alternatively, patients who needed more intensive combined-modality therapy long before referral to the MCLC. The MCLC provides an opportunity to transmit up-to-date information and practice tips about CL to community physicians, including primary care providers (PCP). After each visit, we send a detailed management plan to the patient’s physicians as well as an educational packet with CME programs geared toward our referring physicians to provide resources that enhance their knowledge of the disease process and our treatment recommendations.

### Multimodality clinics as an educational tool

As resident and medical student work hours become more restricted, it is important to maximize the educational benefit in the limited allowable time. For complex or rare medical conditions, a multimodality clinic provides a concentrated patient population and allows the team members to provide collaborative teaching in less time. Because dedicated mentors with extensive knowledge of the disease are intimately involved in patient care, the resident or medical student spends less time on care coordination and more time on education.

With an annual U.S. incidence of only 9.6 cases per 1 million (15), students and residents have limited opportunities to learn about CTCL and other CLs. Over the past 5 years, our clinic has hosted over 50 dermatology and internal medicine residents, 5 dermatology research fellows, 10 dermatopathology fellows, 10 nurse practitioner students, and 120 medical students. Many have commented on their limited exposure to CL throughout their medical school and residency curriculum, further stating that their MCLC experience was a useful tool for intensive learning. The MCLC is a longitudinal practice component of the new OSU Medical School curriculum, called lead, serve, inspire (LSI) (20). LSI serves as a model to discuss the principles and challenges of collaborative research in a course on translational cancer research taught by one of the authors (Pierluigi Porcu) (21). Medical students have emphasized that the highlight of their MCLC rotation was the chance to see exemplary teamwork and cooperation among physicians from different specialties, especially as it relates to longitudinal care for patients with a chronic disease.

### Research in a multimodality clinic

While delivering optimal patient care is the foremost goal of all health centers, academic programs also aim to create and apply new knowledge that improves patient outcomes. The MCLC provides an ideal framework to conduct observational studies spanning the natural history of CLs and allows longitudinal access to clinically annotated biosamples across all stages of the disease to explore prognostic factors and biomarkers. For clinical researchers, the MCLC is simultaneously a source of creative inspiration – a true laboratory of ideas – and the medium and vehicle for the execution of research projects. Firmly wedged in between is the research laboratory, which should be infused with as much clinical insight as possible. Thus, according to the MCLC research model, ideas are often generated in the clinic, explored in the laboratory, and taken back to the clinic. For patients, motivation and interest in research are greatly enhanced by observing how their personal care serves, in real time, as an engine for discovery. Approximately 90% of the MCLC patients give consent for our biorepository and database and, to date, we have archived 2,203 biosamples.

## Conclusion

The multidisciplinary approach to patient care is underutilized in the outpatient setting and its potential in advancing research and education is untested. Obstacles to the implementation of this model include (1) lack of shared outpatient physical space for Dermatology and Hematology–Oncology, (2) billing and documentation that reflect the added value of a multidisciplinary approach, (3) inadequate understanding of its academic worth, (4) access to dedicated disease-specific nursing and supporting staff, and (5) distance from the research laboratory. The analysis presented here is limited but provides preliminary data to support this model. Metrics for performance assessment in the areas discussed are difficult to capture and we constantly seek added insight into how best to manage a multidisciplinary clinic. Health care administrators and practitioners should study and adopt new methodologies to address the added value and unique impact of multimodality clinics, especially in the context of complex diseases.

## Conflict of Interest Statement

The authors declare that the research was conducted in the absence of any commercial or financial relationships that could be construed as a potential conflict of interest.
